# Supplementary material for: An electrochemical biosensor for the detection of Mycobacterium tuberculosis DNA from sputum and urine samples
Source: PLoS One. 2020 Oct 28;15(10):e0241067. doi: 10.1371/journal.pone.0241067 (PMC7592764; doi:10.1371/journal.pone.0241067)
Supplement: S1 Table — (DOCX) [file pone.0241067.s001.docx]

**S1 Table. Table 1.**

Real samples and their respective methods of analysis.

| **Table 1** | | | | | | | |
| --- | --- | --- | --- | --- | --- | --- | --- |
| **SAMPLE** | | **CULTURE - MODS** | | **FAST ACID SMEAR** | | **RESPONSE RATIO** | |
| **MB52** | | Positive | | Negative | | 0.95 | |
| **MB57** | | Positive | | Negative | | 0.92 | |
| **MB255** | | Positive | | Negative | | 0.65 | |
| **MB122** | | Positive | | Negative | | 0.88 | |
| **MB140** | | Positive | | Negative | | 0.9 | |
| **MB141** | | Positive | | Negative | | 0.96 | |
| **MB142** | | Positive | | Negative | | 0.88 | |
| **MB145** | | Positive | | Negative | | 1.16 | |
| **MB147** | | Positive | | Negative | | 0.97 | |
| **MB150** | | Positive | | Negative | | 0.92 | |
| **MB125** | | Positive | | Negative | | 0.81 | |
| **MB265** | | Positive | | Negative | | 0.65 | |
| **MB114** | | Positive | | Negative | | 0.48 | |
| **MB258** | | Positive | | Negative | | 0.92 | |
| **MB200** | | Positive | | Negative | | 0.8 | |
| **MB279** | | Positive | | Negative | | 0.49 | |
| **MB201** | | Positive | | + | | 0.77 | |
| **MB118** | | Positive | | + | | 0.94 | |
| **MB119** | | Positive | | + | | 0.48 | |
| **MB130** | | Positive | | + | | 0.85 | |
| **MB144** | | Positive | | + | | 0.86 | |
| **MB151** | | Positive | | + | | 0.81 | |
| **MB152** | | Positive | | + | | 0.94 | |
| **MB218** | | Positive | | + | | 0.94 | |
| **MB232** | | Positive | | + | | 0.33 | |
| **MB197** | | Positive | | + | | 0.26 | |
| **MB202** | | Positive | | + | | 0.77 | |
| **MB153** | | Positive | | + | | 0.58 | |
| **MB59** | | Positive | | + | | 0.86 | |
| **MB51** | | Positive | | + | | 0.67 | |
| **MB155** | | Positive | | ++ | | 0.58 | |
| **MB129** | | Positive | | ++ | | 0.73 | |
| **M225** | | Positive | | ++ | | 0.59 | |
| **M52** | | Positive | | ++ | | 0.95 | |
| **M199** | Positive | ++ | | 0.33 | |  |  |
| **M144** | Positive | ++ | | 0.26 | |  |  |
| **M191** | Positive | ++ | | 0.49 | |  |  |
| **MB58** | Positive | ++ | | 0.99 | |  |  |
| **MB253** | Positive | ++ | | 0.78 | |  |  |
| **MB102** | Positive | +++ | | 0.88 | |  |  |
| **MB128** | Positive | +++ | | 0.8 | |  |  |
| **MB123** | Positive | +++ | | 0.65 | |  |  |
| **MB154** | Positive | +++ | | 0.89 | |  |  |
| **MB57** | Positive | +++ | | 0.92 | |  |  |
| **MB124** | Positive | +++ | | 0.53 | |  |  |
| **MB157** | Positive | +++ | | 0.89 | |  |  |
| **MB48** | Positive | +++ | | 0.85 | |  |  |
| **M15** | Negative | Negative | | 1.14 | |  |  |
| **M12** | Negative | Negative | | 1.28 | |  |  |
| **M18** | Negative | Negative | | 1.03 | |  |  |
| **M3** | Negative | Negative | | 1.37 | |  |  |
| **M16** | Negative | Negative | | 1.02 | |  |  |
| **M1** | Negative | Negative | | 2.01 | |  |  |
| **M4** | Negative | Negative | | 1.79 | |  |  |
| **M11** | Negative | Negative | | 1.11 | |  |  |
| **M9** | Negative | Negative | | 1.22 | |  |  |
| **M17** | Negative | Negative | | 1.18 | |  |  |
| **M14** | Negative | Negative | | 0.89 | |  |  |
| **M12** | Negative | Negative | | 1.38 | |  |  |
| **M19** | Negative | Negative | | 1.22 | |  |  |
