# Supplementary figures and images for: An electrochemical biosensor for the detection of Mycobacterium tuberculosis DNA from sputum and urine samples
Source: PLoS One. 2020 Oct 28;15(10):e0241067. doi: 10.1371/journal.pone.0241067 (PMC7592764; doi:10.1371/journal.pone.0241067)

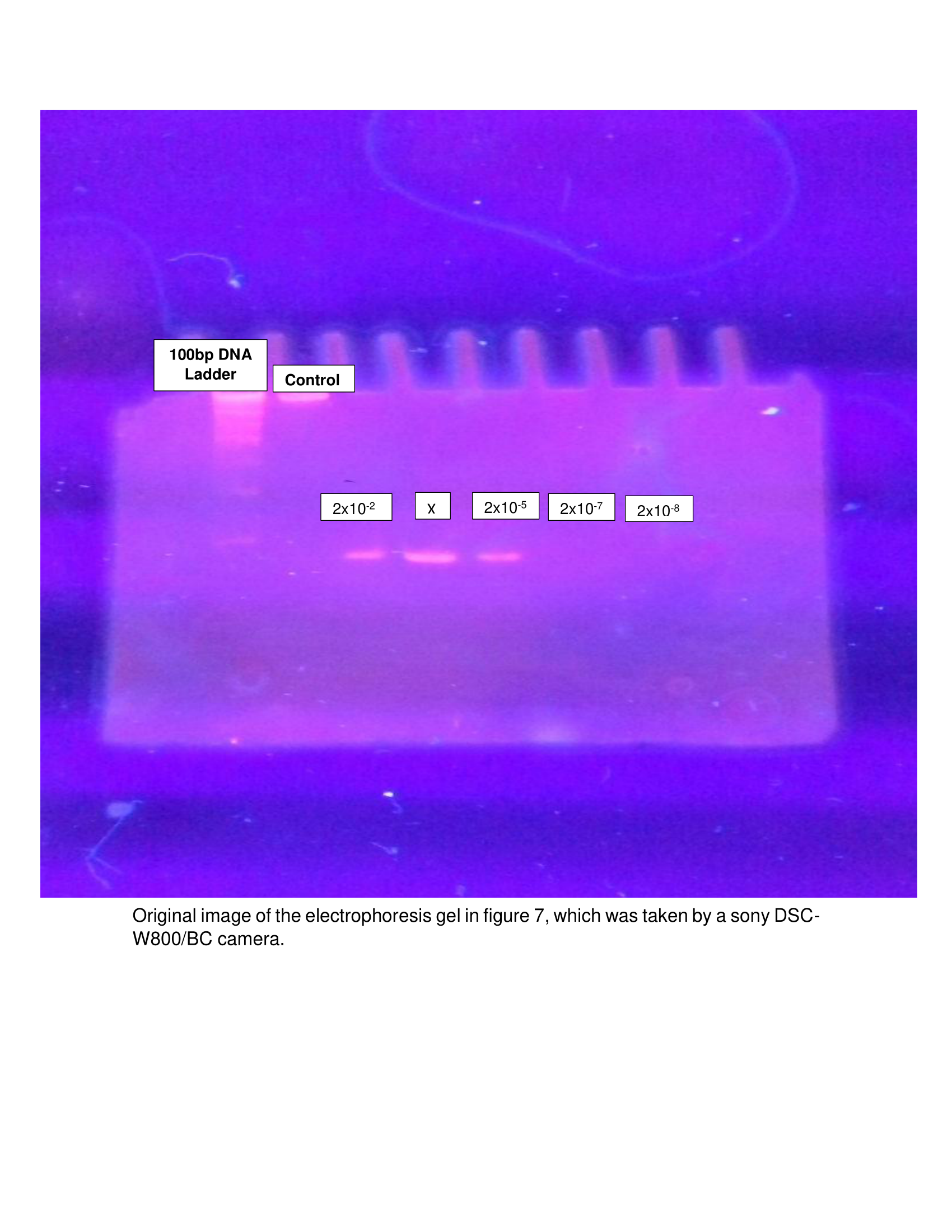

Supplement: S1 Raw images — (TIFF) [file pone.0241067.s002.tiff]
