# Supplementary material for: An electrochemical biosensor for the detection of Mycobacterium tuberculosis DNA from sputum and urine samples
Source: PLoS One. 2020 Oct 28;15(10):e0241067. doi: 10.1371/journal.pone.0241067 (PMC7592764; doi:10.1371/journal.pone.0241067)
Supplement: Attachment — Submitted filename: Response to Reviewers.pdf [file pone.0241067.s003.pdf]

Rebuttal Letter

PONE-D-20-08686
"An electrochemical biosensor for the detection of Mycobacterium tuberculosis DNA from sputum and urine samples"
Daniel Ramos-Sono[1¶], Raúl Laureano[1¶], Daniel Rueda[1], Robert H. Gilman[2], Adolfo La Rosa[3], Jesús Ruiz[4], Raul León[4], Patricia Sheen[1], Mirko Zimic[1*]
PLOS ONE

Jonathan Claussen
Academic Editor
PLOS ONE

Dear Dr. Claussen
Thank you for considering our manuscript for potential publication. Please find below the confirmation of all the actions requested in the editorial comments.

With my best regards,

Mirko Zimic

Major revisions

1. First Paragraph (methods): line 2---Authors state they amplified IS6110 using generic biomarkers. This is vague and explanation could be improved.

Thank you to the reviewer for picking this. We corrected this problem, by explaining it explicitly and more precisely and by adding a new reference in the paragraph of the lines 93 to 97.

> *"We use the insert 6110 (IS6110), which is a specific sequence for the detection of M. tuberculosis DNA. IS6110 is a recognized genotypic biomarker for epidemiology and widely distributed in the strains collected worldwide. Due to its unique presence in Mycobacterium complexes and its high level of replication through the entire genome, IS6110 is used as a gold standard biomarker for diagnosis"*

2. Second paragraph (methods): line 2---Specifying the temp/saline concentrations would be productive to let other labs confirm/repeat experiment.

Thank you to the reviewer for this advice. We definitely promote the replicability of our studies. For this, he conditions requested were added in lines 108 to 110.

“Secondary structure of DNA sequences was evaluated using MFold Web software, with the thermodynamic parameters of 68°C and 10nM for the saline concentration during the amplification process”.

3. Why did the authors use two different concentrations between the first and the second probe (1mM DNA vs 1.5mM DNA). Please explain inconsitency

We thank the reviewer to show this section was not clear and looked inconsistent. Our rationale was that, given that the second probe was smaller in size, if we maintain the same concentration, the surface density would be smaller, and with that, a lower capacity to bind/trap the target DNA. This explanation was added in lines 153 to 156.

“With MiliQ water, dried with N2, and stored at 4ºC. The same procedure was performed with the second capture probe but due to its smaller size (69 pb) a final concentration of 1.5 mM was used, in order to compensate and achieve a similar surface density.”

4. Was the elution buffer used for their DNA purification from urine samples specified?

Thank you. This was corrected. The description of the elution buffer was mentioned in lines 203 and 246.

-203 *“The content of the collection tube was discarded and subsequently added 50 µL of elution buffer (10 mM Tris Buffer, pH 8.5) and was stored at 4°C until it was used.”*

-246 *“The solution was transferred to a silica column for DNA purification and was finally eluted in 50 µL of elution buffer (10 mM Tris Buffer, pH 8.5).”*

5. The determined cutoff value for response ratio seems limited in trials as it was only based on TB positive Bk negative vs TB-negative cultures from this singular sample pool. This could be added to discussion.

Thank you to the reviewer for noticing the importance of this point. To test the sensor in a more adverse scenario, we decided to use TB culture positive and Bk negative samples. In this case, our positive sample will show the lowest concentration of DNA to be detected. We described this in the discussion 394 to 401.

“The sputum samples from TB patients showed a cut-off point of 0.74 response ratio. Furthermore, the sensor reached 97.62% sensitivity and 92.3% specificity, suitable for use it as a diagnostic tool. In the other hand, our method allows the recognition of amplicons from a conventional PCR. In contrast to SDS-PAGE or real time PCR, our technique is free of labels/tags

and dyes that increase the cost of the test. It is important to highlight that the TB culture-positive sputum samples used (N=42), were Bk negative and were contrasted against TB culture negative samples (N=13). This is the most adverse scenario for testing the biosensor, because of the low bacilli load and consequently the low DNA concentration available."

6. First, the data should support the conclusion. For example, how does the author prove the stability and reliability of the method? From the data (Figure 6), there is a lack of experiments and analysis of these two points. The authors should notice that the R-squared value represents the quality of the fit, not the quality of the method. Please mark all data points and errors in Figure 6.

Figure 6 was modified, adding the error bars.

7. The author should clearly explain their method by adding some comparison and analysis of this method with other recognized standard methods.

Thank you for this. We make a clarification of this issue in lines 394 to 401.

"The sputum samples from TB patients showed a cut-off point of 0.74 response ratio. Furthermore, the sensor reached 97.62% sensitivity and 92.3% specificity, suitable for use it as a diagnostic tool. In the other hand, our method allows the recognition of amplicons from a conventional PCR. In contrast to SDS-PAGE or real time PCR, our technique is free of labels/tags and dyes that increase the cost of the test. It is important to highlight that the TB culture-positive sputum samples used (N=42), were Bk negative and were contrasted against TB culture negative samples (N=13). This is the most adverse scenario for testing the biosensor, because of the low bacilli load and consequently the low DNA concentration available."

8 The dynamic range of the test is unclear.
We clarify in line 1 about the dynamic range of our device in the lines 406 to 408

"Moreover, the biosensor using synthetic DNA control tested with urine showed a linear trend in range of 1nM to 100nM, therefore, our biosensor presents a linear dynamic range from 1nM to 100nM in the synthetic sample".

GRAMMATICAL/OTHER MINOR ERRORS:
-**2nd paragraph (intro), 5th line--duration spelled with two "n"s.**
This was corrected in line 67 to 68:

> *"sensitivity, specificity, duration of the test, and operator training being the primary limitations of these techniques."*

-Paragraph 4 (intro): line 2---"electrochemical biosensor based electrochemical detection…" Add "based on".
This was modified in line 87:

> *"sensitive/specific, voltammetric electrochemical biosensor based on electrochemical detection of"*

-Paragraph 4 (intro): line 3---Might be wrong, but after looking it up and looking at other areas of the --paper it seems the authors meant: IS6110 vs IS6610.
It was indeed a typing error, however, this was corrected in the line 88:

> *"DNA hybridization for identification of the DNA sequence IS6110 of Mycobacterium tuberculosis in"*

-inconsistencies in formatting in-text citations. (see paragraph 4 of methods, line 3---used square brackets instead of parenthesis.)
The square brackets was instead of parenthesis in the line 126.

"CTACGTTG3') as capture probe second, previously reported (22). Also, the synthetic sequence 5´-"

-When using their phenol cholorform isoamyl alcohol solution in a sentence they might want to be consistent and keep it phenol:cholorform:isoamyl:alcohol solution.
The use of "phenol:cholorform:isoamyl:alcohol solution" standardized in line 200.

-Bold-face sub-heading "Evaluation of the biosensor with sputum samples from TB patients".
The modification was made in the line 215.

-Citations 16-18 are missing volume numbers and have dates placed twice instead.
The references in lines 532 to 537 will be modified

16. Zhou L, He X, He D, Wang K, Qin D. Biosensing technologies for mycobacterium tuberculosis detection: Status and new developments. Clin Dev Immunol. 2011;2011;19396.
17. Patel S, Nanda R, Sahoo S, Mohapatra E. Biosensors in health care: The milestones achieved in their development towards lab-on-chip-analysis. Biochem Res Int. 2016;2016;31304.
18. Mir M. Oligonucleotide based-biosensors for label-free electrochemical protein and DNA detecction. [Dissertation]. Universitat Rovira i Virgili.; 2007.

-**Update citation 24 to be consistent in formatting (no title/journal volumen**
The reference format in lines 536 to 537 was modified.

18. Mir M. Oligonucleotide based-biosensors for label-free electrochemical protein and DNA detecction. [Dissertation]. Universitat Rovira i Virgili.; 2007.
